# Efficacy of orally administered prednisolone versus partial endodontic treatment on pain reduction in emergency care of acute irreversible pulpitis of mandibular molars: study protocol for a randomized controlled trial

**DOI:** 10.1186/s13063-017-1883-x

**Published:** 2017-03-28

**Authors:** Olivia Kérourédan, Léonard Jallon, Paul Perez, Christine Germain, Jean-François Péli, Dominique Oriez, Jean-Christophe Fricain, Elise Arrivé, Raphaël Devillard

**Affiliations:** 1 CHU de Bordeaux, Pôle d’Odontologie et de Santé Buccale, 33000 Bordeaux, France; 2 INSERM, Bioingénierie Tissulaire, U1026, 33076 Bordeaux, France; 3 Université de Bordeaux, UFR des Sciences Odontologiques, 33082 Bordeaux, France; 4 INSERM, ISPED, Centre INSERM U-897-Epidemiologie-Biostatistique, Bordeaux Cedex, 33076 France; 5 CHU de Bordeaux, USMR, Pôle Santé publique, 33076 Bordeaux, France

**Keywords:** Analgesia, Corticosteroids, Dental emergency, Irreversible pulpitis, Mandibular molars, Pain management, Pulpotomy

## Abstract

**Background:**

Irreversible pulpitis is a highly painful inflammatory condition of the dental pulp which represents a common dental emergency. Recommended care is partial endodontic treatment. The dental literature reports major difficulties in achieving adequate analgesia to perform this emergency treatment, especially in the case of mandibular molars. In current practice, short-course, orally administered corticotherapy is used for the management of oral pain of inflammatory origin. The efficacy of intraosseous local steroid injections for irreversible pulpitis in mandibular molars has already been demonstrated but resulted in local comorbidities. Oral administration of short-course prednisolone is simple and safe but its efficacy to manage pain caused by irreversible pulpitis has not yet been demonstrated. This trial aims to evaluate the noninferiority of short-course, orally administered corticotherapy versus partial endodontic treatment for the emergency care of irreversible pulpitis in mandibular molars.

**Methods/design:**

This study is a noninferiority, open-label, randomized controlled clinical trial conducted at the Bordeaux University Hospital. One hundred and twenty subjects will be randomized in two 1:1 parallel arms: the intervention arm will receive one oral dose of prednisolone (1 mg/kg) during the emergency visit, followed by one morning dose each day for 3 days and the reference arm will receive partial endodontic treatment. Both groups will receive planned complete endodontic treatment 72 h after enrollment. The primary outcome is the proportion of patients with pain intensity below 5 on a Numeric Scale 24 h after the emergency visit. Secondary outcomes include comfort during care, the number of injected anesthetic cartridges when performing complete endodontic treatment, the number of antalgic drugs and the number of patients coming back for consultation after 72 h.

**Discussion:**

This randomized trial will assess the ability of short-term corticotherapy to reduce pain in irreversible pulpitis as a simple and rapid alternative to partial endodontic treatment and to enable planning of endodontic treatment in optimal analgesic conditions.

**Trial registration:**

ClinicalTrials.gov, identifier: NCT02629042. Registered on 7 December 2015.

(Version n°1.1 28 July 2015)

**Electronic supplementary material:**

The online version of this article (doi:10.1186/s13063-017-1883-x) contains supplementary material, which is available to authorized users.

## Background

Irreversible pulpitis is an inflammatory condition of the dental pulp, highly painful, and one of the main reasons for seeking emergency dental treatment [1, 2]. Pain associated with irreversible pulpitis represents more than 45% of the reasons for dental emergency consultation in hospital [3]. Diagnosis of symptomatic irreversible pulpitis is based on clinical findings such as spontaneous mild to severe pain that remains after removal of the stimulus. The most widely used clinical test is the response to heat or cold sensitivity test. The main etiology of irreversible pulpitis is an infectious lesion due to decay or loss of seal under restorations. After tooth trauma, pulp exposure or cracks can also induce a pulpal inflammatory response [4]. Recommended emergency care is partial endodontic treatment under local and/or locoregional anesthesia [5, 6]. The purpose of emergency partial endodontic treatment is to stop the pain of pulpitis by removing a portion of the pulp [7]. Compared to complete pulpectomy, the pulpotomy procedure results in a lower incidence of post-treatment pain [8, 9]. Several dressings can be used after emergency pulpotomies, camphorated phenol, eugenol, isotonic saline and cresatin, without contribution for the relief of pain [7]. Ideally, complete final endodontic treatment is performed in the following 72 h, as 55% of patients experience moderate to severe pain due to pulpotomy [10, 11]. The dental literature reports major difficulty in achieving adequate anesthesia in the mandible in order to perform partial endodontic treatment, especially for molars [12, 13]. This results in a very painful care experience for the patient [14]. Management of this type of emergency is costly for health facilities in terms of equipment and time as pulpotomy is the only emergency treatment recommended [14]. Patient comfort, cost-saving and rationalization of care time justify the search for an alternative to emergency partial endodontic treatment. A recent systematic review by Shirvani et al. [15] showed superior intraoperative analgesia for patients with irreversible pulpitis after administration of preemptive nonsteroidal anti-inflammatory drugs. But, to our knowledge, no clinical trial on the use of orally administered corticosteroid for the treatment of dental pulp inflammation has been conducted. In current practice, short-course, orally administered corticotherapy (prednisolone) is used to manage oral pain of inflammatory origin [16–18]. Glucocorticoids, thanks to their anti-inflammatory action, can neutralize the inflammatory mediators [19]. Pulp inflammation can be treated using this molecule: the efficacy of intraosseous local steroid injection for irreversible pulpitis of mandibular molars has already been demonstrated, but this results in local comorbidities and requires specific materials [20, 21]. Oral administration of short-course prednisolone is simple and safe but its efficacy to manage pain caused by irreversible pulpitis has not yet been demonstrated. Administration of prednisolone per os has a very high (90%) and rapid (at least 4 h) bioavailability. No difference in efficacy between intravenous and oral administration of this molecule was reported in the case of multiple sclerosis [22]. This oral treatment could limit comorbidities and technical difficulties associated with intraosseous injection and could make it possible for complete endodontic treatment to be delayed to 72 h later in optimal conditions of analgesia for the patient. Despite the difficulties described concerning partial endodontic treatment, it is very effective in terms of pain reduction and can achieve a success rate of 100%. A noninferiority design was, therefore, chosen to compare the effect of short-course, orally administered corticotherapy with partial endodontic treatment in terms of pain reduction during adult emergency care for irreversible pulpitis in permanent mandibular molars.

### Objectives

The primary objective of the trial is to compare the effect on pain of short-course, orally administered corticotherapy versus partial endodontic treatment during adult emergency care for irreversible pulpitis in permanent mandibular molars, 24 h after the emergency visit.

The hypothesis is that short-course, orally administered corticotherapy is noninferior to partial endodontic treatment in terms of analgesic efficacy but superior in terms of number of antalgic drugs taken, number of patients coming back to consultation 72 h later, patient comfort and number of injected anesthetic cartridges when performing endodontic treatment.

The secondary objective consists in comparing, depending on the treatment strategy:

- Patient’s comfort during endodontic treatment measured using the “Iowa Satisfaction with Anesthesia Scale” (ISAS) [23]
- Number of analgesic drugs (step 1 on the World Health Organization analgesic ladder or step 2, taken after the inclusion visit and over 72 h)
- Difference in pain measured using the Numeric Scale (NS) between the emergency visit and 24 h thereafter
- Kinetics of pain, self-assessed using the NS at 6, 12, 24, 48 and 72 h after the emergency visit
- Number of injected anesthetic cartridges to achieve absence of pain during complete endodontic treatment
- Number of patients returning for complete endodontic treatment

## Methods/design

The trial protocol was developed in accordance with the Consolidated Standards of Reporting Trials (CONSORT) Statement extension for “Non-Inferiority and Equivalence Trials” [24].

The trial design and protocol adhere to Standard Protocol Items: Recommendations for Interventional Trials (SPIRIT) criteria; the SPIRIT Checklist can be found as Additional file 1: Table S1.

### Design

**Fig. 1:**
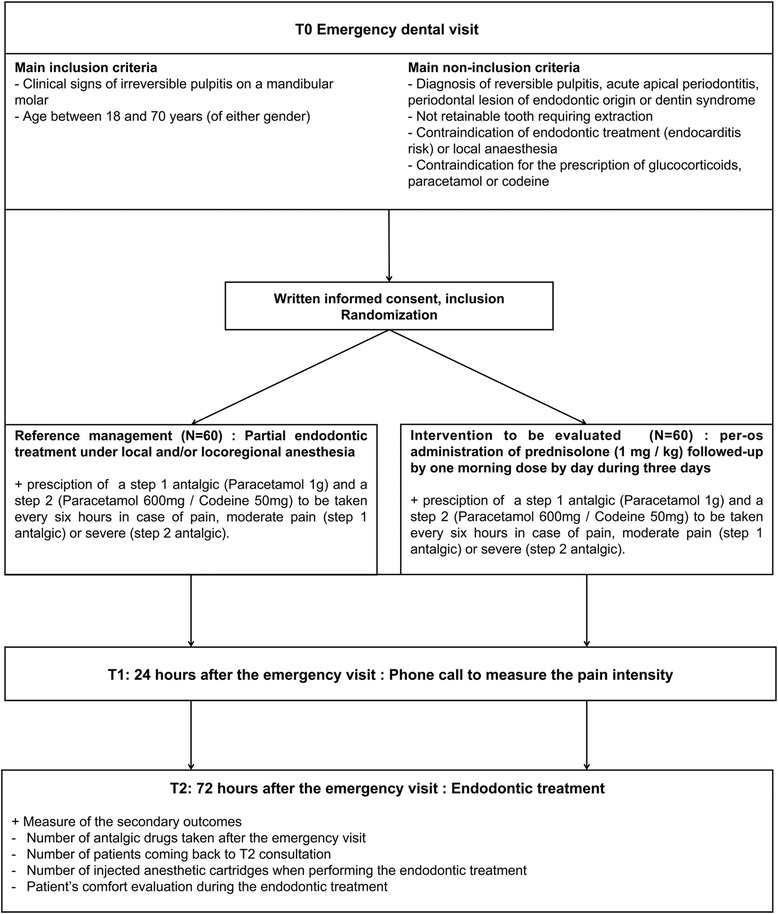
Flowchart of a trial evaluating the noninferiority of short-course, orally administered corticotherapy versus partial endodontic treatment for the pain management in the emergency care of irreversible pulpitis in mandibular molars at the Bordeaux University Hospital

A noninferiority, open-label, randomized controlled clinical trial will be conducted in two dental subunits of the Bordeaux University Hospital. Eligible patients will be recruited during their emergency visit when presenting irreversible pulpitis in the first or second mandibular molars. Diagnosis will be based on clinical and radiographic examination. Two parallel groups will be randomized so that patients will receive: (1) partial endodontic treatment (reference) or (2) short-course, orally administered corticotherapy (intervention to evaluate) (Fig. 1).

#### Participants

Adult patients seeking emergency consultation at the dental department of the teaching hospital in Bordeaux (France) will be recruited if they meet the eligibility criteria. Pulpitis on third molars will be discarded in order to exclude any bias linked to technical difficulties generated by restriction of mouth-opening.

Participants will be included if they meet the following inclusion criteria:

- Clinical signs of irreversible pulpitis of the first or second mandibular molar
- ASA1 or ASA2 score (American Society of Anesthesiologists)
- Aged between 18 and 70 years (of either gender)
- Able to give written informed consent
- Affiliated with a health insurance scheme
- Agree to be contacted by phone 24 h after the emergency visit
- Available to come back 72 h after the emergency visit for complete endodontic treatment

Participants will not be included if they present at least one of the following noninclusion criteria:

- Diagnosis of irreversible pulpitis of the third mandibular molar, reversible pulpitis, acute apical periodontitis, periodontal lesion of endodontic origin or dentin syndrome
- Nonretainable tooth requiring extraction
- Contraindication for endodontic treatment (endocarditis risk), contraindication for the prescription of glucocorticoids or codeine
- Viral disease in evolution (hepatitis, herpes zoster, etc.), machine operators due to the risk of somnolence and lack of attention induced by drugs
- Psychosis uncontrolled by treatment, allergy to one or more of the components
- Immunization with live vaccine
- Diabetes, drug intake with direct interaction with glucocorticoids or codeine, woman of child-bearing age without contraception, pregnant, breastfeeding
- Not able to give informed consent
- Participating in another interventional study

#### Outcomes

The primary outcome is the proportion of patients with pain intensity of below 5 on a Numerical Scale (NS <5) 24 h after the emergency visit [25]. The NS has already been used for the assessment of pain in previous studies to evaluate the efficacy of orally administered corticosteroids on pharyngitis presenting as an emergency [26]. Briefly, the patient will be asked to make a pain rating according to a NS score ranging from 0 to 10. A clinical research assistant will directly phone the patient and will use a standardized sentence: «Please indicate the intensity of your pain level on a scale of 0 (no pain) to 10 (worst pain imaginable)».

Secondary outcomes include:

- Number of analgesic drugs (step 1 on the World Health Organization analgesic ladder (paracetamol 1 g) or step 2 (paracetamol 600 mg/codeine 50 mg) taken after the inclusion visit up to 72 h)
- Difference in pain measured using the NS between T0 (baseline) and 24 h after the emergency visit (T1)
- Kinetics of pain, self-assessed using the NS at 6, 12, 24, 48 and 72 h after T0
- Patient’s comfort during complete endodontic treatment measured using the “Iowa Satisfaction with Anesthesia Scale” (ISAS) [23]
- Number of injected anesthetic cartridges required to achieve clinical silence for the realization of complete endodontic treatment
- Number of patients returning for the T2 visit

### Study timelines

All patients seeking emergency consultation at the dental subunits of the Bordeaux University Hospital will be screened for the selection criteria.

If a patient meets all criteria, they will be informed by an investigator of the study, who will tell them about the objectives, methods, follow-up, risks and restrictions. They will be given an Information Sheet and an Informed Consent Form so that they can read them and ask any questions. If the patient agrees, they will sign the Informed Consent Form and will be included in the study (T0).

**Fig. 2:**
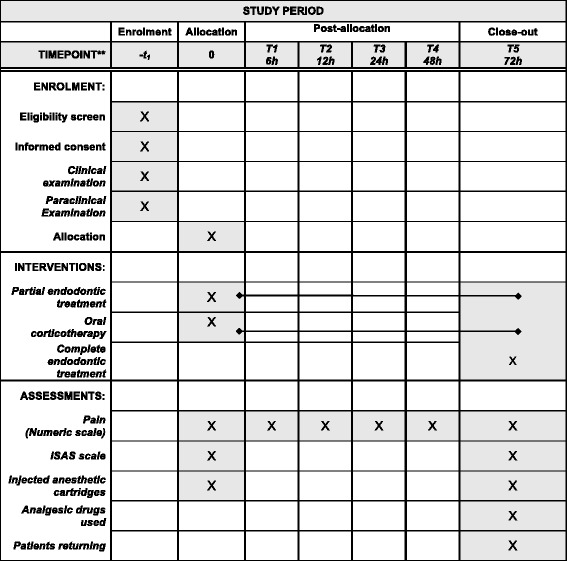
Schedule of enrollment, interventions and assessments during PULPISOLONE

The investigator in charge of the emergency room will proceed to randomization (see below) and another investigator will conduct the T0 (baseline) visit according to the affected group to which the patient will be allocated. The patient will be contacted directly by a research assistant, blinded to the affected group, 24 h after inclusion to gather the information on pain intensity 24 h after the emergency visit (T1). The patient will then receive complete endodontic treatment 72 h after T0 (T2) (Fig. 2).

#### Interventions

The evaluated intervention consists of oral administration of prednisolone (1 mg/kg) during the emergency visit, followed by one morning dose each day for 3 days.

Reference management consists of local and locoregional anesthesia of the molar and partial endodontic treatment. Partial endodontic treatment is a pulpotomy. After preparation and removal of any carious tissue, the tooth will be isolated with a rubber dam and pulpal parenchyma will be removed. Pulpal bleeding will be controlled using 2.5% sodium hypochlorite and the site will be covered with calcium hydroxide and a temporary filling.

At the end of T0, all the patients, whatever their randomization group, will be given two types of antalgics and given the recommendation to take them only if they experience pain. Specifically, it will be recommended to take either a step-1 antalgic (paracetamol 1 g) or a step-2 antalgic (paracetamol 600 mg/codeine 50 mg) every 6 h in case of moderate or severe pain, respectively.

At T2, all patients, will receive full endodontic treatment following local and locoregional anesthesia.

### Randomization

Patients will be randomly assigned to one of the two arms at a ratio of 1:1. The randomization list will be computer-generated by the study statistician using SAS system software (version 9.2, SAS Institute, Inc., Cary, NC, USA). The randomization process will be centralized through a secured website managed by the Bordeaux University Hospital Clinical Trial Unit (CTU) («Unité de Soutien Méthodologique à la Recherche Clinique et Epidémiologique (USMR)»). After confirmation of the patient’s eligibility criteria, the investigator will access the website of the CTU, which will provide the patient’s unique allocation number and randomization group. Access to the final dataset will be limited to the investigators.

### Determination of sample size

Sample size calculation was based on the noninferiority hypothesis that the prescription of short-course corticotherapy would be not less effective than partial endodontic treatment for reducing the pain caused by irreversible pulpitis in mandibular molars.

According to reported results [5], the proportion of patients showing successful relief (presenting pain <5 at 24 h on the NS) in the reference group (partial endodontic treatment) would be 95%. The noninferiority margin was defined as 20% fewer successes in the evaluated group (short-course corticotherapy). The calculation used Newcombe’s formula («lower confidence limit for difference in proportions (simulation)») for the procedure using the 6.0 version of NQuery software and resulted in a sample size of 40 patients per group to achieve 90% power and a one-sided type I error of 0.025.

However, to obtain satisfactory power, two features must be taken into account: (1) a slightly lower proportion of successful treatments in the evaluated group compared to the reference group (power of at least 80% if the treatment group were to have 3% fewer successes or at least 90% if it were to have 2% fewer successes,) and (2) a 5% proportion of patients lost to follow-up or randomly assigned to the corticosteroids group receiving the partial endodontic treatment before the first 24 h due to lack of efficacy [27]. Consequently, it was decided that 60 patients would be enrolled in each group, 120 in total.

### Statistical analysis

The main analysis will be intention-to-treat (ITT) using the «missing = failure» strategy. A robustness analysis, supported by the per-protocol approach, will be carried out.

The difference between the two arms in the number of patients with pain <5 on the NS will be measured using its unilateral 97.5% confidence interval (CI) according to the binomial exact formula. The noninferiority hypothesis will be accepted if the lower confidence limit is superior to the noninferiority margin, which is fixed as −20%.

For qualitative outcomes, frequencies between the two groups will be compared using the chi-square test, or Yates’s corrected chi-square or Fisher’s exact tests, depending on the variables’ distributions. For quantitative outcomes, means will be compared using Student or Wilcoxon tests according to variables’ distributions. Regression model will be used to adjust on major confounding factors (i.e., age and sex). The statistical significance threshold will be 0.05.

Basic statistics in the study report will include information on missing values for all relevant study variables. A summary of baseline patient characteristics with totals and proportions (%) for categorical variables, and minimum, maximum, interquartile ranges and standard deviations for continuous variables will be presented. An estimation of primary and secondary outcomes will be calculated using their 95% CI.

### Protocol violations

All protocol violations occurring after randomization will be listed in the Clinical Study Report, tabulated by subject and recruitment site. The final assignment of participants to the per-protocol analysis will be decided at a blinded protocol violation review meeting before database locking.

### Adverse events

Possible adverse events that may occur during the study will be monitored by investigators and research assistant throughout the study.

## Discussion

The use of short-course, orally administered corticosteroids would allow effective pain management of irreversible pulpitis in mandibular molars. Adverse events of corticosteroids are associated with prolonged intake and high dosing. However, they are not usual in short-term therapy of less than 5 days [16]. Prednisolone is the referent molecule for the short-course treatment of acute and localized inflammation in both medicine generally and stomatology [18, 28].

This new approach in dentistry would increase the number of complete endodontic treatments by avoiding noncompliance of patients because of pain perceived during the emergency visit and improve care and anesthesia according to recommendations of the HAS (French National Authority of Health) [29]. This would reduce trauma related to painful emergency care and thus decrease patient nomadism. It would also reduce material costs and time required when managing irreversible pulpitis in mandibular molars in emergency situations, leading to better rationalization of working time within care structures and decongestion of emergency dental services. Moreover, this treatment can be administered by medical emergency services, which are more accessible and available than dental emergency services.

With this care focused on pain management, it is expected that patients will have a better experience of emergency management and will be more likely to seek further care.

### Trial status

The trial was registered at ClinicalTrials.gov and the study will be open for recruitment in January 2017.

## Additional file

Additional file 1: Table S1. SPIRIT 2013 Checklist for PULPISOLONE. (DOC 121 kb)

## Abbreviations

ASA: American Society of Anesthesiologists
CTU: Clinical Trial Unit
HAS: Haute Autorité de Santé (French National Authority of Health)
ISAS: Iowa Satisfaction with Anesthesia Scale
ITT: Intention-to-treat
NS: Numeric Scale
USMR: Unité de Soutien Méthodologique à la Recherche Clinique et Epidémiologique

## References

[1] Abbott PV, Yu C (2007). A clinical classification of the status of the pulp and the root canal system. Aust Dent J.

[2] Bender IB (2000). Reversible and irreversible painful pulpitides: diagnosis and treatment. Aust Endod J J Aust Soc Endodontology Inc.

[3] Tulip DE, Palmer NOA (2008). A retrospective investigation of the clinical management of patients attending an out of hours dental clinic in Merseyside under the new NHS dental contract. Br Dent J.

[4] Levin LG, Law AS, Holland GR, Abbott PV, Roda RS (2009). Identify and define all diagnostic terms for pulpal health and disease states. J Endod.

[5] Tronstad L. Clinical endodontics: a textbook. 3rd rev. ed. Stuttgart ; New York: Thieme; 2009. 261 p.

[6] Eghbal MJ, Asgary S, Baglue RA, Parirokh M, Ghoddusi J (2009). MTA pulpotomy of human permanent molars with irreversible pulpitis. Aust Endod J J Aust Soc Endodontology Inc.

[7] Hasselgren G, Reit C (1989). Emergency pulpotomy: pain relieving effect with and without the use of sedative dressings. J Endod.

[8] Oguntebi BR, DeSchepper EJ, Taylor TS, White CL, Pink FE (1992). Postoperative pain incidence related to the type of emergency treatment of symptomatic pulpitis. Oral Surg Oral Med Oral Pathol.

[9] Asgary S, Eghbal MJ (2010). The effect of pulpotomy using a Calcium-Enriched Mixture cement versus one-visit root canal therapy on postoperative pain relief in irreversible pulpitis: a randomized clinical trial. Odontology.

[10] Nyerere JW, Matee MI, Simon ENM (2006). Emergency pulpotomy in relieving acute dental pain among Tanzanian patients. BMC Oral Health.

[11] Rapport d’évaluation technologique des traitements endodontiques. Paris: Haute Autorité de Santé; 2008. http://www.has-sante.fr/portail/upload/docs/application/pdf/2009-01/rapport_traitement_endodontique.pdf.

[12] Claffey E, Reader A, Nusstein J, Beck M, Weaver J (2004). Anesthetic efficacy of articaine for inferior alveolar nerve blocks in patients with irreversible pulpitis. J Endod.

[13] Aggarwal V, Singla M, Kabi D (2010). Comparative evaluation of effect of preoperative oral medication of ibuprofen and ketorolac on anesthetic efficacy of inferior alveolar nerve block with lidocaine in patients with irreversible pulpitis: a prospective, double-blind, randomized clinical trial. J Endod.

[14] Carrotte P (2004). Endodontics: Part 3 Treatment of endodontic emergencies. Br Dent J.

[15] Shirvani A, Shamszadeh S, Eghbal MJ, Marvasti LA, Asgary S (2017). Effect of preoperative oral analgesics on pulpal anesthesia in patients with irreversible pulpitis—a systematic review and meta-analysis. Clin Oral Investig.

[16] Holte K, Kehlet H (2002). Perioperative single-dose glucocorticoid administration: pathophysiologic effects and clinical implications. J Am Coll Surg.

[17] Alexander RE, Throndson RR (2000). A review of perioperative corticosteroid use in dentoalveolar surgery. Oral Surg Oral Med Oral Pathol Oral Radiol Endod.

[18] Klossek J-M, Desmonts-Gohler C, Deslandes B, Coriat F, Bordure P, Dubreuil C (2004). Treatment of functional signs of acute maxillary rhinosinusitis in adults. Efficacy and tolerance of administration of oral prednisone for 3 days. Presse Médicale Paris Fr 1983.

[19] Hargreaves KM, Costello A (1990). Glucocorticoids suppress levels of immunoreactive bradykinin in inflamed tissue as evaluated by microdialysis probes. Clin Pharmacol Ther.

[20] Gallatin E, Reader A, Nist R, Beck M (2000). Pain reduction in untreated irreversible pulpitis using an intraosseous injection of Depo-Medrol. J Endod.

[21] Isett J, Reader A, Gallatin E, Beck M, Padgett D (2003). Effect of an intraosseous injection of Depo-Medrol on pulpal concentrations of PGE2 and IL-8 in untreated irreversible pulpitis. J Endod.

[22] Burton JM, O’Connor PW, Hohol M, Beyene J (2012). Oral versus intravenous steroids for treatment of relapses in multiple sclerosis. Cochrane Database Syst Rev.

[23] Peñarrocha-Oltra D, Ata-Ali J, Oltra-Moscardó M-J, Peñarrocha-Diago M, Peñarrocha M. Side effects and complications of intraosseous anesthesia and conventional oral anesthesia. Med Oral Patol Oral Cirugia Bucal. 2011. http://www.ncbi.nlm.nih.gov/pubmed/22143716. Accessed 22 Apr 2012. 10.4317/medoral.17512 PMC3476103 22143716

[24] Piaggio G, Elbourne DR, Pocock SJ, Evans SJW, Altman DG, CONSORT Group (2012). Reporting of noninferiority and equivalence randomized trials: extension of the CONSORT 2010 statement. JAMA.

[25] Jensen MP, Turner LR, Turner JA, Romano JM (1996). The use of multiple-item scales for pain intensity measurement in chronic pain patients. Pain.

[26] Bulloch B, Kabani A, Tenenbein M (2003). Oral dexamethasone for the treatment of pain in children with acute pharyngitis: a randomized, double-blind, placebo-controlled trial. Ann Emerg Med.

[27] Piantadosi. Clinical trials: a methodologic perspective. In: Probability and statistics. New York: Wiley; 1997. p. 175–7.

[28] Société francophone de medecine buccale et de chirurgie buccale. Recommandations pour la prescription des anti-inflammatoires en chirurgie buccale chez l’adulte. Med Buccale Chir Buccale. 2008;14:129–59.

[29] European Society of Endodontology (2006). Quality guidelines for endodontic treatment: consensus report of the European Society of Endodontology. Int Endod J.

